# Comparative Efficacy and Safety of Bariatric Surgery and Bariatric Endoscopy for Obesity Management: A Network Meta-Analysis

**DOI:** 10.1055/a-2892-8092

**Published:** 2026-06-30

**Authors:** Jena Velji-Ibrahim, Dhruvil Radadiya, Harsh Patel, Cesare Hassan, Nicola Pugliese, Kalpit Devani, Prateek Sharma

**Affiliations:** 1 368074 University of South Carolina School of MedicineGreenvilleSouth CarolinaUnited States; 2 Department of Internal Medicine 3626 Prisma Health UpstateGreenvilleSouth CarolinaUnited States; 3 Section of Gastroenterology and Hepatology 12279 Wake Forest University School of MedicineWinston-SalemNorth CarolinaUnited States; 4 Department of Gastroenterology 22494 Cedars-Sinai Medical CenterLos AngelesCaliforniaUnited States; 5 Department of Gastroenterology, Hepatology and Motility 12251 The University of Kansas School of MedicineKansas CityKansasUnited States; 6 Department of Biomedical Sciences 437807 Humanitas UniversityPieve Emanuele, MilanItaly; 7 Endoscopy Unit 9268 IRCCS Humanitas Research HospitalRozzano, MilanItaly; 8 Division of Internal Medicine and Hepatology, Department of Gastroenterology 9268 IRCCS Humanitas Research HospitalRozzano, MilanItaly; 9 Department of Gastroenterology and Liver Center 3626 Prisma HealthGreenvilleSouth CarolinaUnited States; 10 Department of Gastroenterology, Hepatology and Motility 21638 The University of Kansas Medical CenterKansas CityKansasUnited States; 11 Department of Gastroenterology 20044 Kansas City VA Medical CenterKansas CityMissouriUnited States

**Keywords:** quality and logistical aspects, performance and complications, endoscopy upper GI tract

## Abstract

**Background and aims** While bariatric surgery has proven effective for obesity management, its high cost and associated risks have led to increasing interest in alternatives such as bariatric endoscopy. We compared the efficacy and safety of bariatric surgery and bariatric endoscopy for obesity management in adults with and without type 2 diabetes mellitus (T2DM).

**Methods** A comprehensive database search identified randomized controlled trials comparing bariatric surgery and endoscopy in adults with a BMI of 30–45 kg/m ^2^ and at least 52 weeks of follow-up. The primary endpoint was percentage total body weight loss (%TBWL). Secondary endpoints included HbA1c reduction, T2DM remission, and safety. A frequentist network meta-analysis with a random-effects model was conducted.

**Results** In all, 15 RCTs comprising 1548 adults were included. We included four bariatric surgeries [Roux-en-Y gastric bypass (RYGB), laparoscopic sleeve gastrectomy (LSG), greater curvature plication (GCP), and laparoscopic adjustable gastric banding (LAGB)] and three bariatric endoscopies [endoscopic sleeve gastroplasty (ESG), primary obesity surgery endoluminal (POSE), and intragastric balloon (IGB)]. RYGB resulted in the greatest %TBWL (−19.39%), followed by LSG (−12.63%), ESG (7.74%), and POSE (5.62%). RYGB, LSG, and ESG were associated with significant reductions in HbA1c, while ESG and RYGB also achieved significant T2DM remission. Bariatric endoscopy, particularly ESG (OR 4.65; 95% CI: 0.49 to 44.47) and POSE (OR 1.06; 95% CI: 0.48 to 2.34), demonstrated a favorable safety profile, with fewer serious adverse events compared to bariatric surgery (OR 8.40; 95% CI: 1.29 to 54.78). However, overlap in the 95% confidence intervals for both efficacy and safety outcomes limits the certainty of comparative conclusions across interventions.

**Conclusions** Bariatric endoscopy offers a favorable safety profile and clinically meaningful weight loss, representing a minimally invasive treatment option for obesity management and metabolic improvement.

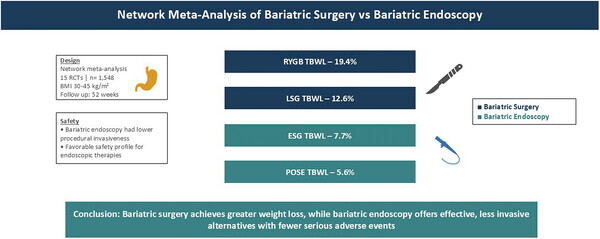

## Introduction

Obesity is a global epidemic, with significant socioeconomic impact. Since 1975, the prevalence of obesity worldwide has tripled. 1 The World Health Organization estimates that more than 600 million people worldwide are obese and 1.9 billion people are overweight. 1 The increasing prevalence of obesity is contributing to the global healthcare burden including type 2 diabetes (T2DM), dyslipidemia, hypertension, metabolic dysfunction-associated steatotic liver disease, certain cancers, and obstructive sleep apnea. 1 2

Efforts to reverse the obesity epidemic encompass a spectrum ranging from lifestyle modifications like dietary changes and physical activity to the more intensive intervention of bariatric surgery. Bariatric surgery has proven to be effective not just in achieving but also in maintaining substantial weight loss over a prolonged period, as well as notably improving comorbidities. 3 4 However, it is associated with perioperative mortality and long-term adverse events. 5 Moreover, only 1% of eligible patients undergo surgery, with patient preference being a substantial factor in its underutilization. 6 7 This leaves most without sufficient obesity treatment.

Studies have shown that even a 5% reduction in total body weight can yield various health benefits, including improvement or prevention of metabolic disease. 8 9 10 Recent guidelines extend eligibility to patients with a BMI ≥ 35 kg/m ^2^ or ≥ 30 kg/m ^2^ with metabolic diseases such as type 2 diabetes mellitus (T2DM). Bariatric endoscopy has evolved into a safe and effective minimally invasive solution to bridge this treatment gap. 11 12 13 This encompasses various procedures including endoscopic sleeve gastroplasty (ESG), intragastric balloon (IGB), and primary obesity surgery endoluminal (POSE). While weight loss medications such as glucagon-like peptide-1 receptor agonists (GLP-1 RAs) have attracted increased attention, recent findings underscore the cost-effectiveness of bariatric endoscopy over GLP-1 RAs. ESG, for example, has demonstrated promising weight loss outcomes, and in a single short-term study, it was associated with a 3-fold lower cost compared to semaglutide. 14

While randomized controlled trials (RCTs) have been conducted to compare bariatric surgery and bariatric endoscopy, they have typically been limited to the comparison of laparoscopic sleeve gastrectomy (LSG) to ESG, have had a limited sample size, and results have been inconsistent. Meta-analyses have evaluated bariatric interventions, including both surgical and endoscopic approaches; however, many have incorporated observational data, heterogeneous study designs, or a limited range of clinical endpoints. Therefore, a comprehensive study encompassing RCTs of bariatric surgery and endoscopic therapies is lacking. This systematic review and network meta-analysis is aimed to investigate the weight loss efficacy and safety profile of bariatric surgery and endoscopy in adults with obesity with and without concurrent T2DM.

## Methods

### Data Sources

Embase, MEDLINE, and Cochrane were systematically searched from inception to March 1st, 2025 for trials assessing bariatric surgery and bariatric endoscopy treatment efficacy and safety in adults with obesity, both with and without T2DM. The protocol for this study was registered on PROSPERO (CRD42024539689). Search terms included keywords such as "obesity", "overweight", "bariatric surgery", "metabolic bariatric surgery", "bariatric endoscopy", as well as the specific names of bariatric surgeries and bariatric endoscopy interventions for computer-assisted literature search ( **eTable 1** ). We restricted the search to clinical trials published in the English language and involving human subjects.

### Study Selection

Our inclusion criteria for clinical trials were as follows: (1) RCTs comparing bariatric surgeries or bariatric endoscopies with lifestyle intervention/medical therapy (nonsurgical therapy) or with each other; (2) duration of 52 weeks; (3) participants aged 18 years or older; (4) participants with a BMI of between 30 and 45 kg/m ^2^ ; and (5) primary or secondary outcome of change in total body weight. We excluded studies in specific populations including patients with type 1 diabetes, diabetes secondary to a specific disease or glucocorticoid therapy, liver disease, renal dysfunction, previous bariatric or major gastrointestinal surgery, pregnancy, or inflammatory disease of the gastrointestinal tract.

### Data Extraction

The primary outcome of this network meta-analysis was efficacy – percentage total body weight loss (%TBWL). This was selected as the primary outcome given its consistent reporting across both surgical and endoscopic interventions and its clinical relevance as a standardized measure of treatment response. The secondary outcomes were safety: gastrointestinal side effects (nausea, vomiting, and abdominal pain) or device-related or procedure-related serious adverse events requiring surgical, endoscopic, or radiological intervention. We also evaluated the impact on HbA1c as well as T2DM remission.

### Quality Assessment

Two reviewers (J.V. and D.R.) independently extracted data from the RCTs and documented it on a standardized data-collection form, including study name and year, sample size, efficacy and safety outcomes of interest, and follow-up period. Baseline patient characteristics such as age, sex, baseline body weight, BMI, waist circumference, and HbA1c were also recorded. Discrepancies were resolved by a third author (K.D.). Two authors (J.V. and D.R.) independently assessed the risk of bias using the Cochrane Risk of Bias Tool Version 2 15 and quality was assessed using the JADAD scale. 16 The third author (K.D.) was consulted in case of any conflicts in the assessment.

### Data Synthesis and Analysis

For this network meta-analysis, we utilized R software. 17 The mean difference (MD) was applied for continuous outcomes and odds ratios (OR) for dichotomous outcomes, along with 95% confidence intervals (95% CI). Direct pairwise comparison was performed using the DerSimonian–Laird random-effects model. 18 When continuous variables were presented as median, range, or interquartile range, they were converted to mean and standard deviation (SD) using the “estmeansd” R package. 19 For dichotomous variables, a continuity correction of 0.5 was used in the setting of zero-cell counts. Statistical heterogeneity was measured using *I* ^2^ statistics. 20 We performed random-effects frequentist network meta-analysis to analyze the direct and indirect comparisons of therapeutic approaches using R “netmeta” package. 21 A generalized Cochrane Q test was used to assess network homogeneity. 22 The generalized Cochrane Q score was further split into within-design and between-design heterogeneity scores to identify contributions to generalized heterogeneity in the random effects model. 23 Publication bias was assessed using a comparison-adjusted funnel plot and Egger’s test. 24 Publication bias in the case of network meta-analysis indicates the possibility of a new therapeutic approach with a small sample reporting positive outcomes being selectively published. All reported *p* -values are two-sided, with statistical significance set at *p* < 0.05. This meta-analysis was reported in accordance with the Preferred Reporting Items for Systemic Reviews and Meta-analyses (PRISMA) statement 25 and its extension for network meta-analysis (PRISMA-NMA). 26

The assumptions of transitivity were considered by evaluating the similarity of included studies with respect to key clinical and methodological variables, including baseline BMI, study duration, and comparator definitions. Given the expected clinical and methodological heterogeneity across trials, a random-effects model was selected a priori.

### Subgroup Analysis

We performed a network meta-analysis in patients with and without T2DM to confirm our results in the general population suffering from obesity without diabetes.

## Results

**Fig. 1:**
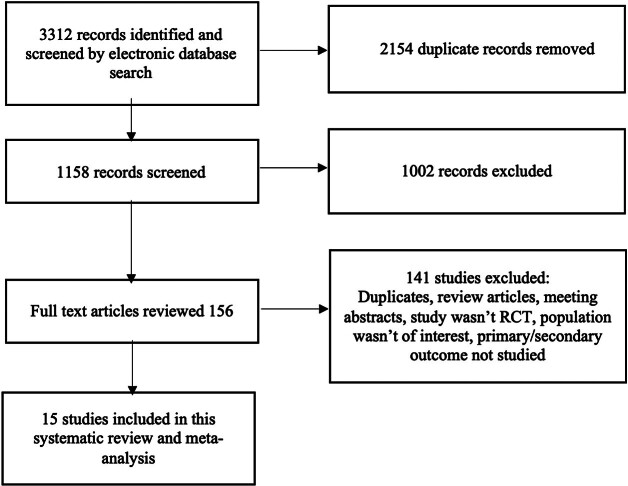
Flowchart summarizing search strategy for study selection.

The study selection process is shown in Fig. 1 . This study included 15 RCTs with sample sizes ranging from 29 to 332 participants. All trials were open-label. Six trials compared an intervention with lifestyle modifications, 27 28 29 30 31 32 one trial compared an intervention to medical therapy, 33 five trials compared an intervention to a combination of lifestyle and medical therapy, 34 35 36 37 38 two compared one intervention to another, 39 40 and one compared an intervention with both lifestyle/medical treatment and other interventions. 41 Medical therapy was based on guidelines from the American Diabetes Association or the European Association for the Study of Diabetes and included treatments such as metformin and insulin. 42 **eTable 2** provides a summary of the included studies in this NMA. Overall, this analysis included 1,548 subjects with obesity with a BMI of 30–45 kg/m ^2^ . Participant ages ranged from 38 to 55 years on average, with 29.4% male participants. Baseline characteristics for body weight, BMI, and HbA1c are detailed in **eTable 2** .

**Fig. 2:**
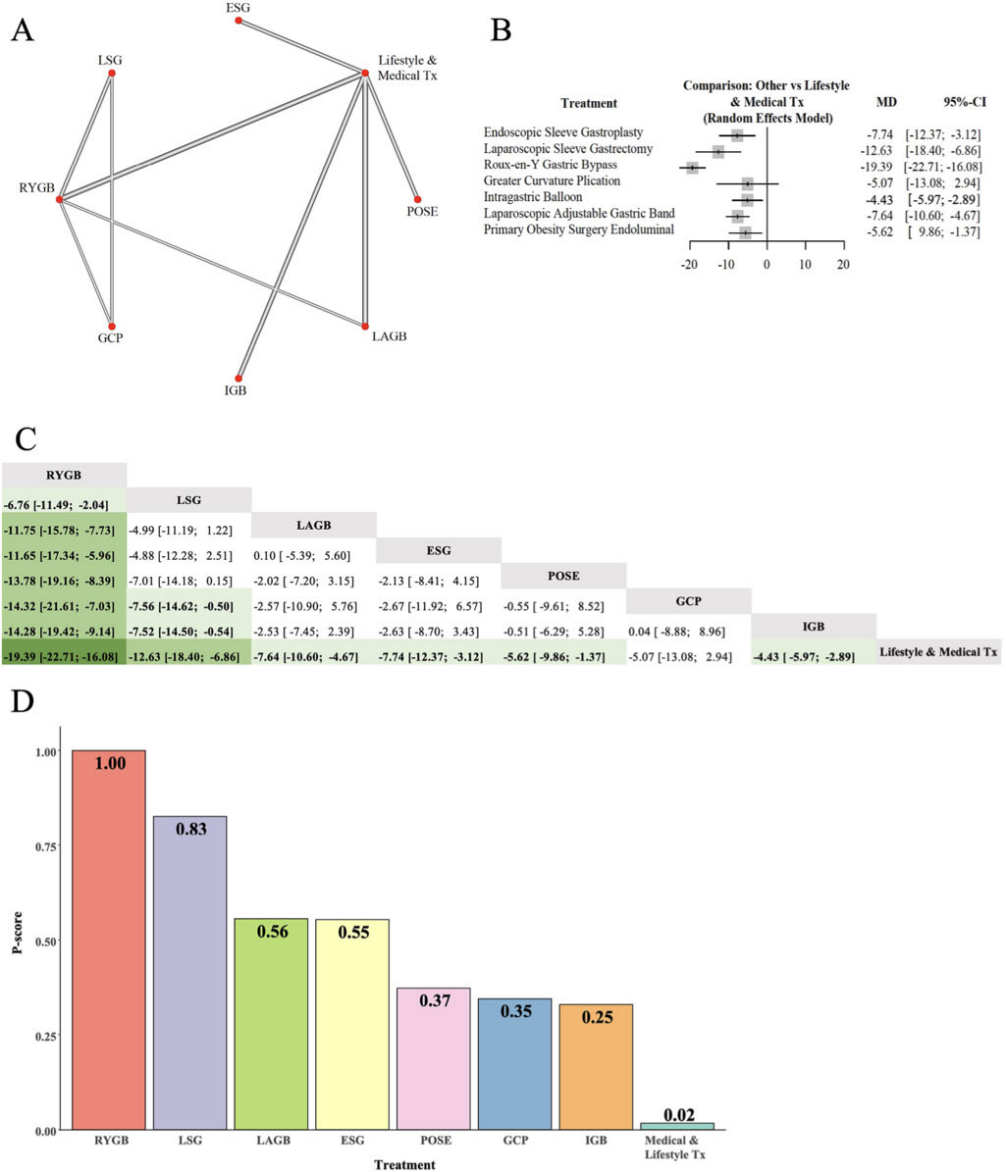
Comparison of weight reduction among endoscopic interventions and bariatric surgeries. ( **A** ) Exhibits the network graph with 7 different interventions, excluding lifestyle and medical treatment. ( **B** ) Illustrates a forest plot with network comparison using a random effects model. It shows that all interventions were associated with a reduction in %TWL. RYGB had the largest effect. ( **C** ) Displays the league table of direct and indirect comparisons of %TWL by intervention. ( **D** ) Contains a rankogram, which identifies the preferred intervention for %TWL.

We included four bariatric surgeries [RYGB, LSG, greater curvature plication (GCP), and laparoscopic adjustable gastric banding (LAGB)] and three bariatric endoscopic interventions (ESG, POSE, and IGB). Fig. 2A shows our overall network plot representing the interventions compared in this NMA.

### Primary Outcome

#### Percentage Change in Body Weight

When compared with lifestyle intervention/medical therapy, all interventions were associated with a reduction in percentage total body weight, as shown in Fig. 2B . RYGB had the largest reduction in %TBWL when compared to nonsurgical therapy (MD: −19.39%; 95% CI: −22.71% to −16.08%, Fig. 2B ). Moreover, RYGB was associated with significant weight loss compared to all other bariatric surgeries and bariatric endoscopic interventions. %TBWL was 12.63% with LSG, 7.74% with ESG, and 5.62% with POSE ( Fig. 2B ). However, there was no statistical difference in %TBWL between LSG and ESG (MD −4.88%; 95% CI −12.28% to 2.51%, Fig. 2C ). In addition, there was no statistical difference between LSG and POSE (MD −7.01%; 95% CI −14.18% to 0.15%, Fig. 2C ). LSG was associated with significant weight loss compared to IGB (MD −7.52%; 95% CI −14.50% to −0.54%, Fig. 2C ). When all treatments were ranked by P-score, RYGB had the highest probability of being the first-ranked treatment for weight loss at 100%, followed by LSG (83%) ( Fig. 2D ). Direct pairwise meta-analyses compared %TBWL with one intervention versus another or lifestyle/medical therapy ( **eFig. 6** ).

### Secondary Outcomes

#### HbA1c Change

**Fig. 3:**
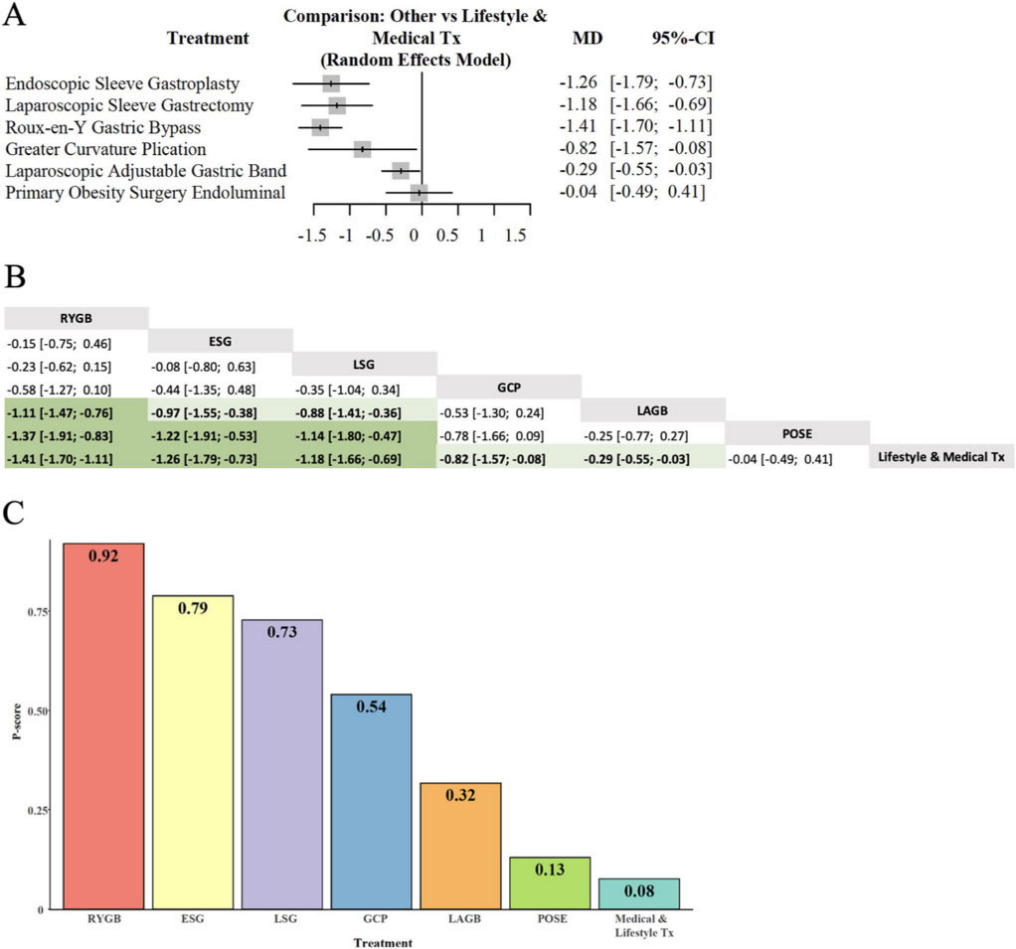
Comparison of HbA1c reduction among endoscopic interventions and bariatric surgeries. ( **A** ) Exhibits a forest plot of HbA1c reduction with network comparison using a random effects model. ( **B** ) Displays the league table of direct and indirect comparisons of HbA1c reduction by intervention. ( **C** ) Contains a rankogram, which identifies the preferred intervention for HbA1c reduction.

Compared to nonsurgical therapy, RYGB, LSG, LAGB, GCP, and ESG were associated with a significant reduction in HbA1c ( Fig. 3A ). POSE was not associated with a significant HbA1c reduction. Of all endoscopic interventions, only ESG was associated with a significant HbA1c reduction. RYGB, LSG, and ESG were all associated with a significant HbA1c reduction compared with LAGB and POSE ( Fig. 3B ). There was no significant difference between RYGB, LSG, GCP, and ESG. RYGB had the highest probability of being ranked first for mean HbA1c reduction (92%), followed by ESG (79%) and LSG (73%) ( Fig. 3C ). Direct pairwise meta-analyses compared changes in HbA1c between interventions and lifestyle/medical therapy ( **eFig. 7** ).

#### Diabetes Remission

**Fig. 4:**
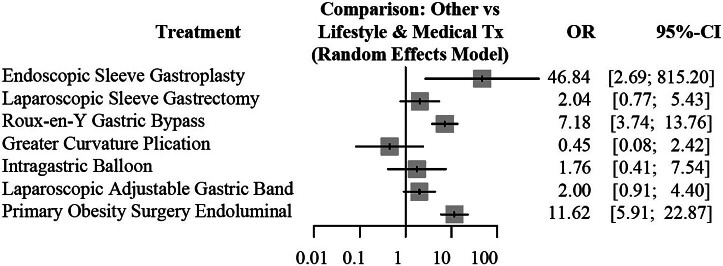
Comparison of T2DM remission among interventions.

Compared to medical therapy/lifestyle intervention, ESG, RYGB, and POSE were associated with T2DM remission ( Fig. 4 ). LSG, GCP, IGB, and LAGB were not associated with diabetes remission when compared to nonsurgical treatment. Diabetes remission was compared between different interventions with each other and lifestyle/medical therapy using direct pairwise meta-analyses ( **eFig. 8** ). The number of patients with T2DM remission was small in several trials, which may restrict the interpretation of T2DM remission.

#### Safety

**Fig. 5:**
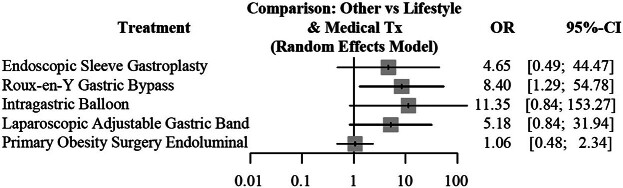
Comparison of side effect profile among interventions, exhibiting a forest plot with network comparison using a random effects model.

Serious adverse events reported by RCTs included postprocedure abdominal pain, nausea, and vomiting, procedure reversal, leak, ulcer or abscess formation, and bleeding. All procedures were more likely to be associated with side effects compared to medical therapy/lifestyle intervention, except for POSE. ESG, POSE, and LAGB had a more favorable safety profile, with no increased odds of mortality, intensive care needs, or surgery compared to RYGB and IGB ( Fig. 5 ). **eFig. 9** illustrates direct pairwise meta-analyses.

#### Publication Bias and Network Coherence

Visual evaluation of funnel plot symmetry and quantitative analysis of small study effects (Egger’s regression test, *P* > 0.05) suggested no evidence of publication bias for the network meta-analysis except for the analysis of T2DM remission ( **eFigs. 1–4** ). Network heterogeneity and coherence were assessed using generalized Cochran’s *Q* statistics, including within- and between-design components. Overall, there was no significant inconsistency detected across the network, although clinical and methodological heterogeneity between studies should be considered when interpreting results ( **eFig. 5** ).

#### Risk of Bias and Quality Assessment

All trials except for Hofso et al. 40 were open-label. In several trials, the description of blinding of assessors and attrition rate were inadequate. Risk of bias assessment with the ROB2 tool showed that the majority of the studies were of low risk of bias. Quality assessment using the JADAD score showed that all RCTs had a score of more than 4 2 ( **eTable 3)** .

## Discussion

In this systematic review and network meta-analysis encompassing 15 RCTs with 1,548 adults, we compared bariatric surgery and bariatric endoscopy for obesity management. We demonstrate that all interventions were associated with weight reduction. RYGB achieved the greatest %TBWL (19.39%), followed by LSG (12.63%), ESG (7.74%), and POSE (5.62%). There was no significant difference in %TBWL between LSG and bariatric endoscopy including ESG and POSE. When compared to nonsurgical therapy, RYGB, LSG, GCP, LAGB, and ESG were associated with significant HbA1c reduction. Of all bariatric endoscopic interventions, only ESG was associated with a significant HbA1c reduction and diabetes remission. Bariatric endoscopy, including ESG and POSE, had a better side effect profile, with no increased odds of mortality, intensive care needs, or surgery compared to bariatric surgery.

RCTs have compared bariatric surgeries to each other. We show that the %TBWL was 19.39 with RYGB and 12.63 with LSG. While RYGB had a greater reduction in HbA1c and T2DM remission than LSG, the difference between the two was not significant. This aligns with previous meta-analyses highlighting a greater efficacy of RYGB compared to LSG for BMI, glycemic control, and lipid profile. 43 44 While few RCTs have directly compared other surgical interventions, previous meta-analysis has demonstrated that LAGB has a similar efficacy and safety profile to LSG. We describe similar results as %TBWL with LAGB was 7.64, with no significant difference compared to LSG. Conversely, GCP appears to be less effective, consistent with existing literature. 43

Endoscopic interventions, including ESG, POSE, and IGB, were associated with statistically significant weight loss. Both ESG and POSE involve reshaping the stomach to limit its capacity, whereas IGB entails placing a balloon in the stomach to induce the sensation of fullness. Studies have demonstrated that ESG and POSE produce greater weight loss and improve comorbidities such as T2DM when compared to IGB. The multicenter MERIT trial illustrated the efficacy of ESG in the management of obese patients. 27 Sharaiha and colleagues illustrated a reduction in HbA1c, systolic blood pressure, ALT, and triglycerides at one year with ESG. 45 For POSE, a previous meta-analysis that included both observational studies and RCTs showed a %TBWL. 12 The MILEPOST trial demonstrated a difference in satiety at 12 months between the POSE procedure group and the lifestyle modifications group. 28 The randomized, sham-controlled ESSENTIAL trial found that POSE achieved 3.6 times better %TBWL as compared with placebo. 29 Our results are similar, illustrating a significant weight loss with ESG (7.74%) and POSE (5.62%). Of the bariatric endoscopic interventions we included, IGB produced the least weight loss. Major complications of IGB include balloon migration and intolerance and a major limitation of IGB is the weight regain after the removal of the balloon. 46 Overall, while ESG and POSE were associated with greater weight loss compared to IGB, the magnitude of weight loss remained lower than that observed with bariatric surgical interventions. These findings suggest that bariatric endoscopy may serve as an effective minimally invasive treatment option, particularly for patients who are not candidates for or prefer to avoid surgery.

Serious adverse events cannot be ignored. The principal adverse effects of bariatric surgeries include anastomotic leaks, bleeding, re-operations, and vitamin deficiencies. 43 Bariatric endoscopy has been associated with bleeding, GERD, postprocedure abdominal pain, nausea, and vomiting. 12 13 47 48 We found that ESG and POSE had a more favorable side effect profile compared to IGB, RYGB, and LAGB, although comparative estimates included wide confidence intervals. A meta-analysis of 15 studies demonstrated that while the %TBWL associated with LSG was 30.5 and ESG was 17.1, the pooled rate of adverse events associated with LSG was 11.8% compared to 2.9% for the ESG group. 47 This is consistent with our findings of ESG having a superior side effect profile to LSG. Other studies revealed that the incidence of serious adverse events and early removal after IGB was higher than the incidence of serious adverse events for POSE, consistent with our analysis. 12 49 However, long-term data on the safety of bariatric surgery and endoscopy are needed. Additionally, data on overweight individuals and those >65 years old are lacking.

Pharmacotherapy provides an alternative obesity management strategy, and GLP-1 RAs have undoubtedly reshaped the landscape of obesity management, offering a pharmacologic approach with notable weight-reduction benefits. However, despite their efficacy, long-term weight loss outcomes with GLP-1 RAs may not match those achieved through endoscopic therapy. Bariatric endoscopy has emerged as a promising alternative, often yielding substantial and sustained weight loss. Moreover, the cost-effectiveness of bariatric endoscopy presents a significant advantage over both GLP-1 agonists and bariatric surgery. Haseeb and colleagues’ economic modeling study in patients with class II obesity found that ESG was more cost-effective than semaglutide over a 5-year horizon, offering greater weight loss and a $33,583 reduction in total cost; however, these findings are based on modeled projections and require confirmation with long-term clinical data. 14 Endoscopic techniques offer the benefits of being less invasive compared to surgery, thereby reducing associated risks. Additionally, the lower costs associated with bariatric endoscopy make it an attractive option for individuals seeking effective and economical solutions for obesity management. When compared to LSG, ESG was shown to be more cost-effective with fewer complications and reoperations, 50 more cost-effective over a 5-year period despite higher upfront costs, 14 and resulted in shorter hospital stays. 51 Consequently, while GLP-1 RAs have revolutionized obesity management, bariatric endoscopy presents a compelling alternative, providing both significant weight loss outcomes and enhanced cost-effectiveness compared to both pharmaceutical and surgical interventions.

To our knowledge, this is one among the first comprehensive studies synthesizing exclusively RCT data to comprehensively compare bariatric surgery to bariatric endoscopy across multiple clinically relevant endpoints, including %TBWL, HbA1c, T2DM remission, and safety. While prior network meta-analyses have evaluated bariatric interventions, many have incorporated observational data, focused on individual procedural classes, or did not directly compare bariatric surgery and endoscopy within a unified randomized framework. Our study provides complementary evidence with a focus on internal validity and clinical applicability. A few head-to-head trials compared one type of bariatric surgery to another, typically comparing RYGB and LSG. The RCTs investigating bariatric endoscopy have been limited to comparing one intervention to placebo. While we show that RYGB did produce the most weight loss, we demonstrate the efficacy of bariatric endoscopy—ESG, POSE, and IGB for obesity management. Importantly, we show that bariatric endoscopy has a superior side effect profile compared to bariatric surgery. With guidelines qualifying patients for bariatric interventions having a BMI of 30 kg/m ^2^ , this offers patients the flexibility to choose an intervention based on their preference.

Several important limitations should be considered when interpreting these findings. Although this network meta-analysis was restricted to RCTs, the included studies varied in baseline participant characteristics including BMI and trial duration. To improve comparability, we limited inclusion to RCTs with participants having a BMI between 30 and 45 kg/m ^2^ and a duration of 52 weeks to standardize results. Subgroup analysis based on BMI (e.g., <40 vs ≥40 kg/m ^2^ ) was not feasible due to the limited number of bariatric endoscopy trials including patients with BMI ≥ 40, in contrast to bariatric surgery studies that more frequently enrolled this population. The duration of T2DM and baseline glycemic control also varied between studies. Most trials did not report outcomes stratified by insulin use, oral hypoglycemic therapy, or individualized metabolic surgery score, limiting adjustment for baseline diabetes severity. Additionally, some studies were single-center with a small sample size, and attrition in some trials may have affected the results. The definition of nonsurgical therapy varied and consisted of either lifestyle modifications, pharmacotherapy, or both, depending on the trial. Notably, GLP-1 RAs were not included in this network meta-analysis due to the absence of sufficient head-to-head RCTs directly comparing these agents with both surgical and endoscopic interventions within a unified analytical framework. Technical heterogeneity across endoscopic platforms may have influenced pooled estimates, particularly for ESG, as included studies utilized different suturing platforms and allowed crossover in some trials, potentially attenuating long-term between-group differences. In addition, the POSE studies reflected earlier fundus-targeting techniques that are no longer routinely performed. To date, no randomized controlled trials evaluating distal POSE (POSE 2.0) were identified in our search, limiting the applicability of these findings.

The available evidence remains limited by relatively small sample sizes and overlapping 95% confidence intervals, which reduced statistical power to detect differences between interventions and precludes definitive conclusions regarding superiority between interventions. This may in part be due to the relatively small number of RCTs across a large number of interventions. The wide confidence intervals combined with limited sample sizes may explain the lack of an observed difference between LSG and POSE despite differences in their reported %TBWL. Notably, the pooled %TBWL observed in this analysis was lower than that reported in large observational and registry-based studies. This discrepancy may reflect the use of conservative intention-to-treat analyses, stricter eligibility criteria, standardized follow-up protocols, shorter follow-up duration, and limited use of adjunctive therapies in randomized trials, which may yield more modest weight loss estimates compared with real-world data. Furthermore, follow-up duration was standardized to 52 weeks for network comparability but precluded the assessment of long-term durability and weight regain. Publication bias was detected for studies reporting T2DM remission ( **eFig. 3** , *p* -value from Egger’s test is 0.0163), and when combined with small sample sizes and short follow-up, these results should be interpreted with caution. Additionally, safety outcomes incorporated minor and severe adverse events which may have introduced heterogeneity and limited the comparability of pooled safety estimates across interventions. Collectively, these limitations highlight the need for larger, well-powered trials with longer follow-up and contemporary procedural techniques to better define the comparative effectiveness of bariatric surgical and endoscopic interventions.

In conclusion, in this network meta-analysis of randomized controlled trials, we demonstrate that bariatric endoscopy, including ESG, POSE, and IGB, represents a promising minimally invasive treatment option that may help bridge the gap in obesity management for patients who are not candidates for or prefer to avoid surgery. Given their significant weight loss and superior side effect profile, minimally invasive endoscopic procedures appear to be effective treatment options for patients with obesity who cannot or choose not to undergo bariatric surgery. Future RCTs are needed for long-term efficacy and safety outcomes, the assessment of subpopulations such as those who are >65 years old and with class I obesity, as well as the comparison of bariatric surgery and endoscopy to GLP-1 agonists for obesity management. Studies of combined therapies such as bariatric endoscopy with GLP-1 agonists as an effective alternative to bariatric surgery should be explored. Additionally, comparing the impact of these procedures on the improvement of medical comorbidities will further elucidate the differences between these bariatric endoscopy techniques.

## Supplementary Material

Ergänzendes Material Supplementary Material
