# Supplementary material for: Comparative Efficacy and Safety of Bariatric Surgery and Bariatric Endoscopy for Obesity Management: A Network Meta-Analysis
Source: Endosc Int Open. 2026 Jun 30;14:a28928092. doi: 10.1055/a-2892-8092 (PMC13334204; doi:10.1055/a-2892-8092)

eTable 1: Search strategy.

**Pubmed**<br>(("bariatric surgery"[MeSH Terms] OR ("bariatric"[All Fields] AND "surgery"[All Fields]) OR "bariatric surgery"[All Fields] OR ("metabolic"[All Fields] AND "surgery"[All Fields]) OR "metabolic surgery"[All Fields]) OR ("bariatric surgery"[MeSH Terms] OR ("bariatric"[All Fields] AND "surgery"[All Fields]) OR "bariatric surgery"[All Fields])) AND (("obesity"[MeSH Terms] OR "obesity"[All Fields]) OR ("diabetes mellitus"[MeSH Terms] OR ("diabetes"[All Fields] AND "mellitus"[All Fields]) OR "diabetes mellitus"[All Fields] OR "diabetes"[All Fields])) AND (Randomized Controlled Trial[ptyp] AND "humans"[MeSH Terms])<br><br>(("endoscopic intervention"[MeSH Terms] OR ("endoscopic"[All Fields] AND "intervention"[All Fields]) OR "endoscopic bariatric treatment"[All Fields] OR ("endoscopic"[All Fields] AND ("bariatric"[All Fields] AND "treatment"[All Fields]) OR "endoscopic therapy"[All Fields]) OR ("endoscopy"[MeSH Terms] AND "therapy"[All Fields]) OR "endoscopic therapy"[All Fields])) AND (("obesity"[MeSH Terms] OR "obesity"[All Fields]) OR ("diabetes mellitus"[MeSH Terms] OR ("diabetes"[All Fields] AND "mellitus"[All Fields]) OR "diabetes mellitus"[All Fields] OR "diabetes"[All Fields])) AND (Randomized Controlled Trial[ptyp] AND "humans"[MeSH Terms])
**EMBASE**<br>'bariatric surgery' AND 'obesity' AND ('obesity'/exp OR obesity) AND [embase]/lim NOT ([embase]/lim AND [medline]/lim) AND 'randomized controlled trial'/de<br><br>'endoscopic intervnetion' AND 'obesity' AND ('obesity'/exp OR obesity) AND [embase]/lim NOT ([embase]/lim AND [medline]/lim) AND 'randomized controlled trial'/de
**Cochrane**<br>1 "obesity"[MeSH Terms] OR "overweight"[MeSH Terms] OR "body mass index"[MeSH Terms] OR ("body"[All Fields] AND "mass"[All Fields] AND "index"[All Fields]) OR "obesity"[All Fields] or "overweight"[All Fields]<br>2 "diabetes mellitus"[MeSH Terms] OR ("diabetes"[All Fields] AND "mellitus"[All Fields] OR "diabetes mellitus"[All Fields]<br>3 "diabetes mellitus, type 2"[MeSH Terms] OR "type 2 diabetes mellitus"[All Fields] OR "type 2 diabetes"[All Fields]<br>4 1 OR 2 OR 3<br>5 "bariatric surgery"[MeSH Terms] OR ("bariatric"[All Fields] AND "surgery"[All Fields]) OR "bariatric surgery"[All Fields]<br>6 "metabolic bariatric surgery"[MeSH Terms] OR ("metabolic"[All Fields] AND ("bariatric"[All Fields]" AND surgery"[All Fields]) OR "metabolic bariatric surgery"[All Fields]<br>7 "metabolic surgery"[MeSH Terms] OR ("metabolic"[All Fields] AND surgery"[All Fields]) OR "metabolic surgery"[All Fields]<br>8 5 OR 6 OR 7

9	"endoscopic intervention"[MeSH Terms] OR ("endoscopic"[All Fields] AND "intervention"[All Fields]) OR "endoscopic intervention"[All Fields]
10	"endoscopic bariatric treatment"[MeSH Terms] OR ("endoscopic"[All Fields] AND "bariatric"[All Fields] AND "treatment"[All Fields]) OR "endoscopic bariatric treatment"[All Fields]
11	"endoscopic therapy"[MeSH Terms] OR ("endoscopic"[All Fields] AND "therapy"[All Fields]) OR "endoscopic therapy"[All Fields]
12	9 OR 10 OR 11
13	4 AND 8
14	4 AND 12
15	13 AND Clinical Trial[ptyp]
16	14 AND Clinical Trial[ptyp]

Additional search:
Additional manual search of the references of included trials and former meta-analyses was carried out to identify other newly published and unpublished studies.

eTable 2. Baseline characteristics of participants from studies included in our network meta-analysis.

STUDY		ABU DAYYEH, 2022		CASAJOANA, 2021			HUBERTY, 2020		SIMONSON, 2019		HOFSO, 2019		IKRAMUDDIN, 2018		SIMONSON, 2018	
**INTERVENTION/COMPARATOR**		Control	ESG	RYGB	LSG	GCP	Control	ESG	Control	LAGB	RYGB	LSG	Control	RYGB	Control	RYGB
**SAMPLE SIZE, N**		124	85	15	15	15	22	49	22	18	54	55	56	57	19	19
**AGE, YEARS**		45.7	47.3	51	49.2	49.7	45.3	37.6	51.6	51	48.2	47.1	48	49	52.6	50.7
**SEX, MALE %**		18	12	53.3	66.7	80	9	6	59	50	26	42	45	35	47	32
**BASELINE BODY WEIGHT, KG**		99.1	98.4	103	102	105	94.7	93.3	111.6	106.8	124.4	126.7	99	98	102.7	104.6
**BMI, KG/M^2**		35.7	35.5	38.7	39	40.7	34.2	34.8	36.7	6.4	42.4	42.1	34.4	34.9	36.5	36
**WAIST CIRCUMFERENCE, CM**		109.7	110.3	-	-	-	-	-	114.4	115.9	127	128	114	114	114.1	117.8
**HBA1c %**		5.8	5.8	7.39	7.89	8.05	-	-	8.08	8.41	7.6	7.9	9.6	9.6	8.78	8.24
**BLOOD PRESSURE, MMHG**	SBP	131.6	134.2	-	-	-	-	-	126.2	128.5	131	132	132	127	126	132
	DBP	80.9	82.4	-	-	-	-	-	80.9	79.1	84	84	79	78	77	82
**CHOLESTEROL, MG/DL**	Total	192	193.8	-	-	-	-	-	160.6	55.3	170	178	186	181	162	154
	HDL	54.9	54.5	-	-	-	-	-	42	36.7	97	99	41	41	39	44
	LDL	111.9	115	-	-	-	-	-	91.5	92.3	39	40	102	102	99	88
**TRIGLYCERIDES, MG/DL**		123.3	120.8	-	-	-	-	-	145	176	198	195	211	200	156	120

STUDY		XIANG, 2018		MILLER, 2017		COURCOULAS, 2017		SULLIVAN, 2016		CUMMINGS, 2016		COURCOULAS, 2015			DING, 2015	
**INTERVENTION/COMPARATOR**		Control	LAGB	Control	POSE	Control	IGB	Control	POSE	Control	RYGB	Control	RYGB	LAGB	Control	LAGB
**SAMPLE SIZE, N**		44	44	10	34	130	125	111	221	17	15	20	20	21	22	23
**AGE, YEARS**		51	47	38.5	38.3	40.8	38.7	45.3	44.2	55	52	47	47	47	51	51
**SEX, MALE %**		21	23	10	26	10	10	9	11.8	41	20	18	18	18	59	39
**BASELINE BODY WEIGHT, KG**		96.1	97.5	96.8	99.9	98	98	98.7	99.7	112.8	108.8	102	99	100	111	107
**BMI, KG/M^2**		35	35.7	37.2	36.2	35	35	36.2	36	37	38	36	36	36	36.7	36.4
**WAIST CIRCUMFERENCE, CM**		-	-	-	-	-	-	108.5	108.5	120.8	121.7	112	111	115	-	-
**HBA1c %**		5.8	5.9	-	-	-	-	5.6	5.6	7.3	7.7	7.0	8.6	7.9	8.1	8.4
**BLOOD PRESSURE, MMHG**	SBP	126	127	-	-	126	126	133	132	120	129	132	140	135	126	129
	DBP	76	78	-	-	80	80	79	78	75	77	76	81	77	81	79
**CHOLESTEROL, MG/DL**	Total	174	174	-	-	195	192	195	199	170	166	182	200	190	161	155
	HDL	46	46	-	-	54	52	52	51	43	43	44	42	40	42	37
	LDL	104	104	-	-	117	113	119	123	85	93	106	118	91	91	92
**TRIGLYCERIDES, MG/DL**		109	117	-	-	-	-	121	130	204	151	161	170	222	145	176

STUDY		FULLER, 2013	
INTERVENTION/COMPARATOR		Control	IGB
SAMPLE SIZE, N		35	31
AGE, YEARS		48	43
SEX, MALE %		34	32
BASELINE BODY WEIGHT, KG		103	105
BMI, KG/M^2		36.7	36
WAIST CIRCUMFERENCE, CM		115.2	115.4
HBA1c %		-	-
BLOOD PRESSURE, MMHG	SBP	130	134
	DBP	83	86
CHOLESTEROL, MG/DL	Total	197	217
	HDL	45	48
	LDL	120	132
TRIGLYCERIDES, MG/DL		168	195

eTable 3. Jadad score results for all trials.

	Abu Dayyeh., 2022	Casajoana, 2021	Huberty, 2020	Simonson, 2019	Hofso, 2019	Ikramuddin, 2018	Simonson, 2018	Xiang, 2018	Miller, 2017	Courcoulas, 2017	Sullivan, 2016	Cummings, 2016	Courcoulas, 2015	Ding, 2015	Fuller, 2013
Described as randomized*	1	1	1	1	1	1	1	1	1	1	1	1	1	1	1
Described as double-blind*	0	0	0	0	1	0	0	0	0	0	1	0	0	0	0
Description of withdrawals	1	1	1	1	1	1	1	1	1	1	1	1	1	1	1
Randomization method described and appropriate*	1	1	1	1	1	1	1	1	1	1	1	1	1	1	1
Double-blinding method described and appropriate	1	1	1	1	1	1	1	1	1	1	1	1	1	1	1
**Score**	**4**	**4**	**4**	**4**	**5**	**4**	**4**	**4**	**4**	**4**	**5**	**4**	**4**	**4**	**4**

eFigure 1. Comparison adjusted funnel plot of total body weight loss across included studies. The symmetry of the plot suggests no major publication bias.

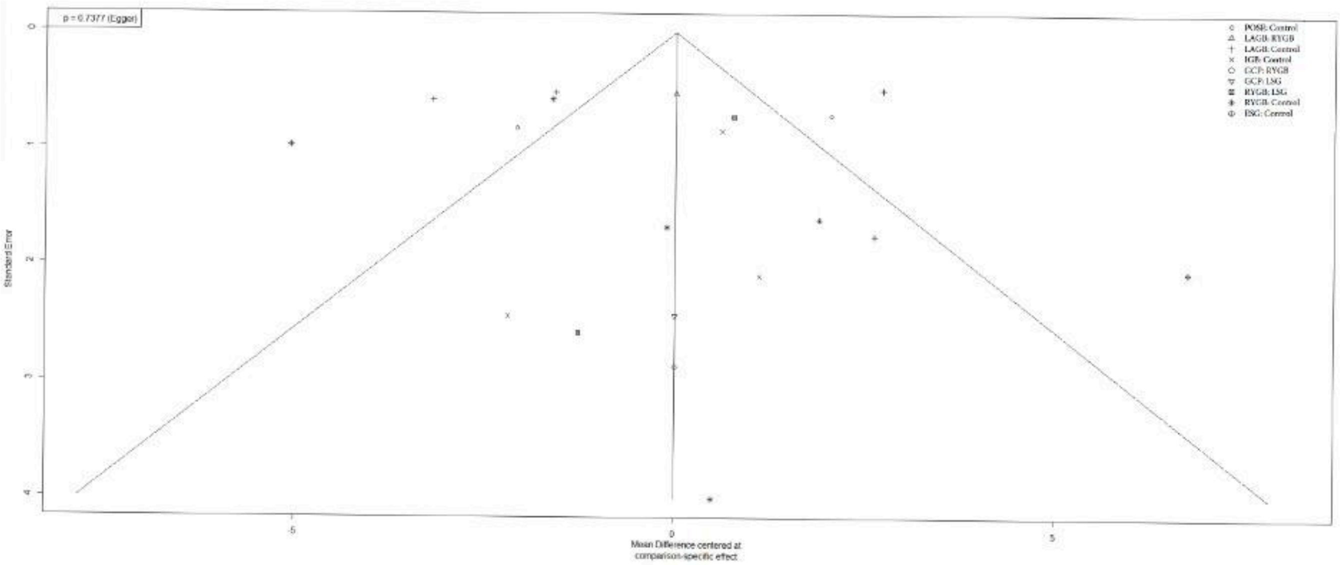

eFigure 2. Funnel plot of HbA1c from RCTs comparing one intervention to another or control. Visual assessment shows symmetry, indication no strong evidence of publication bias.

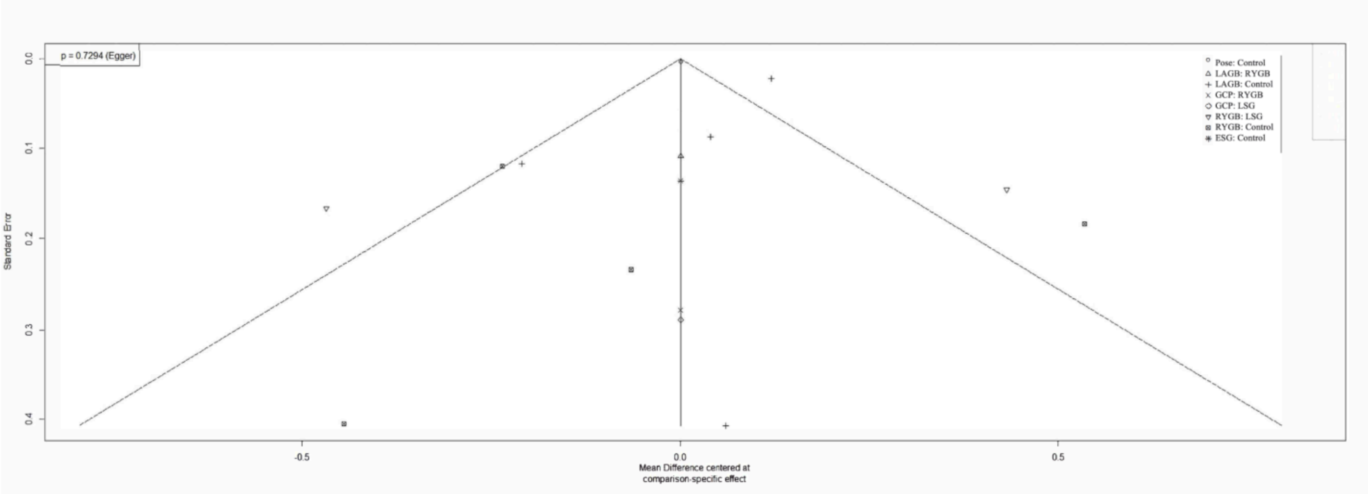

eFigure 3. Funnel plot of T2DM remission of studies comparing one intervention to another or control. Here, p-value from Egger's test is 0.0163 (two-sided p-value).

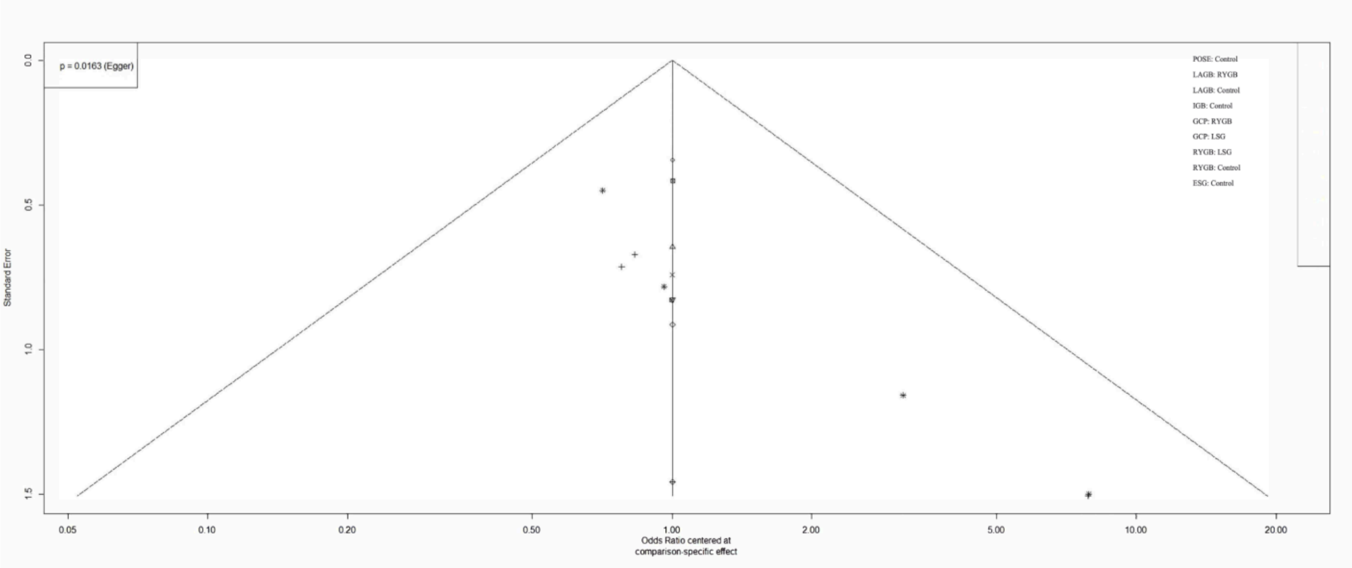

eFigure 4. Funnel plot of serious adverse events from studies comparing one intervention to another or control. Visual inspection suggests symmetry, consistent with a low likelihood of publication bias.

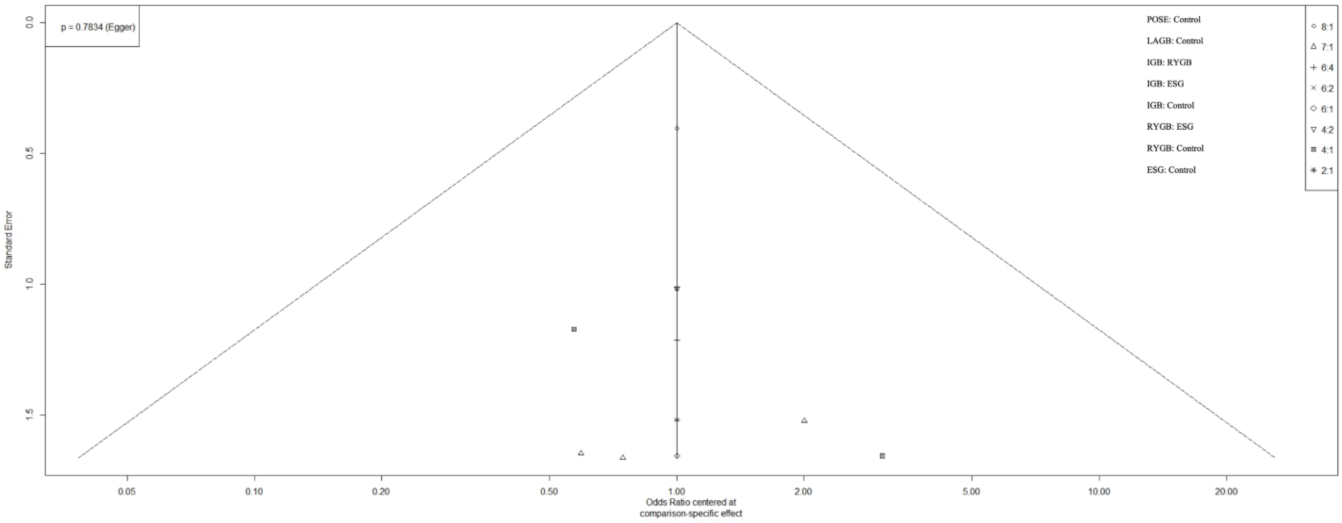

eFigure 5. Risk of bias summary. 5A includes the risk of bias assessment using Cochrane Risk of Bias 2 tool. 5B illustrates each risk of bias item presented as percentages across all included

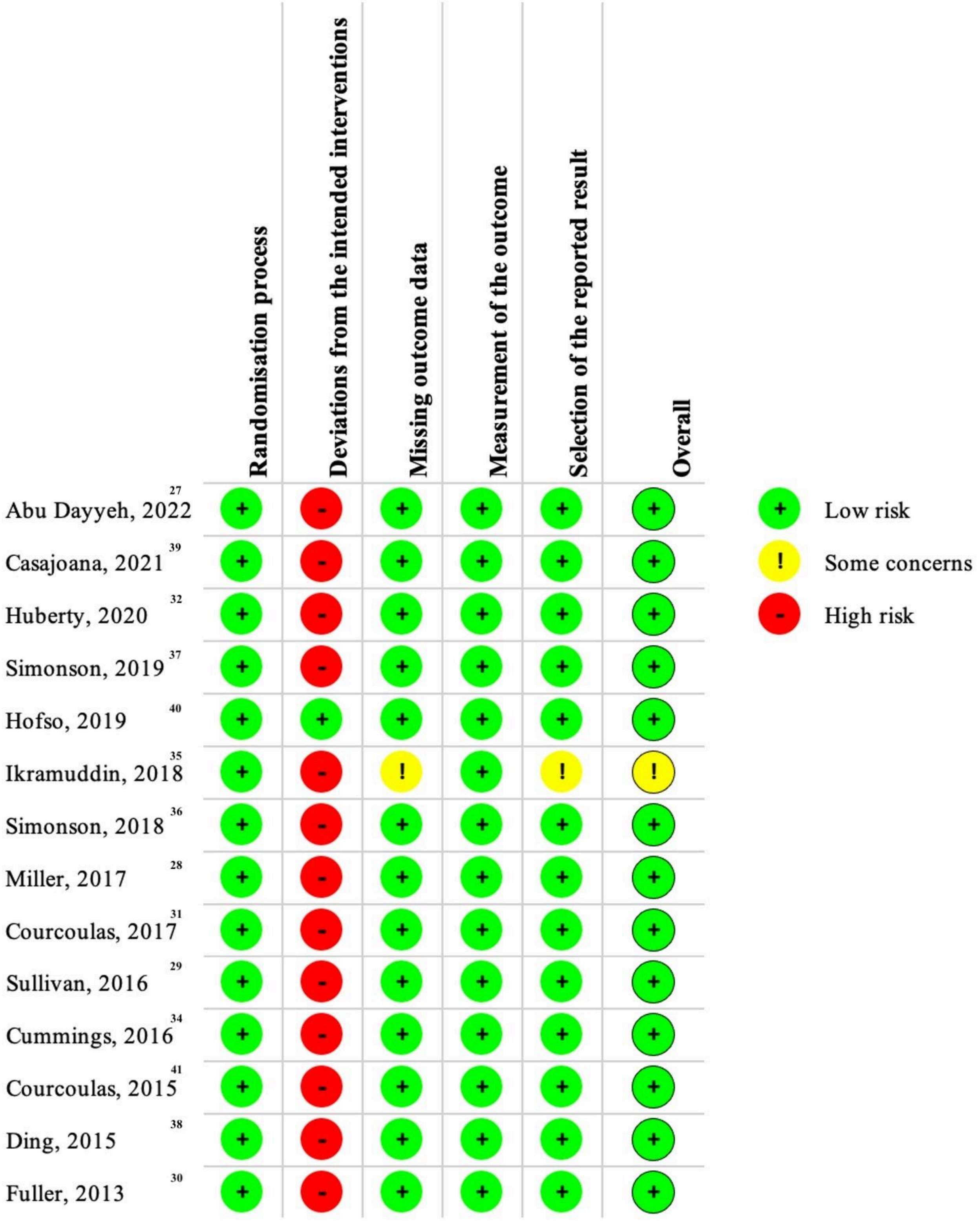

studies.

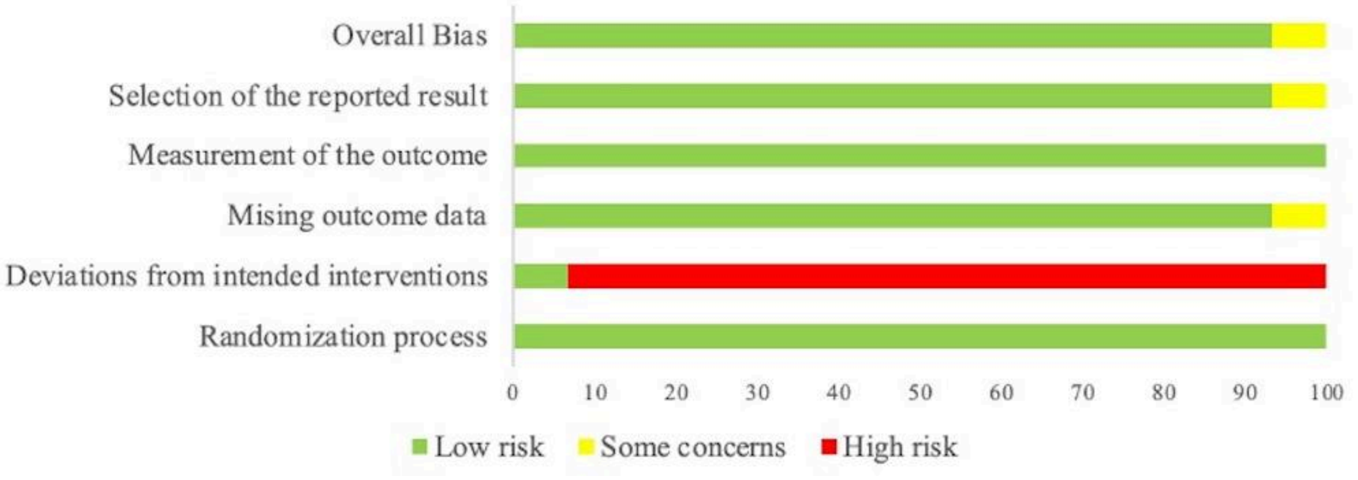

Endosc Int Open | DOI 10.1055/a-2892-8092 | © The Author(s).

Thieme

eFigure 6. Direct pairwise comparisons of total body weight loss with one intervention versus another or control at endpoint using a random effects model.

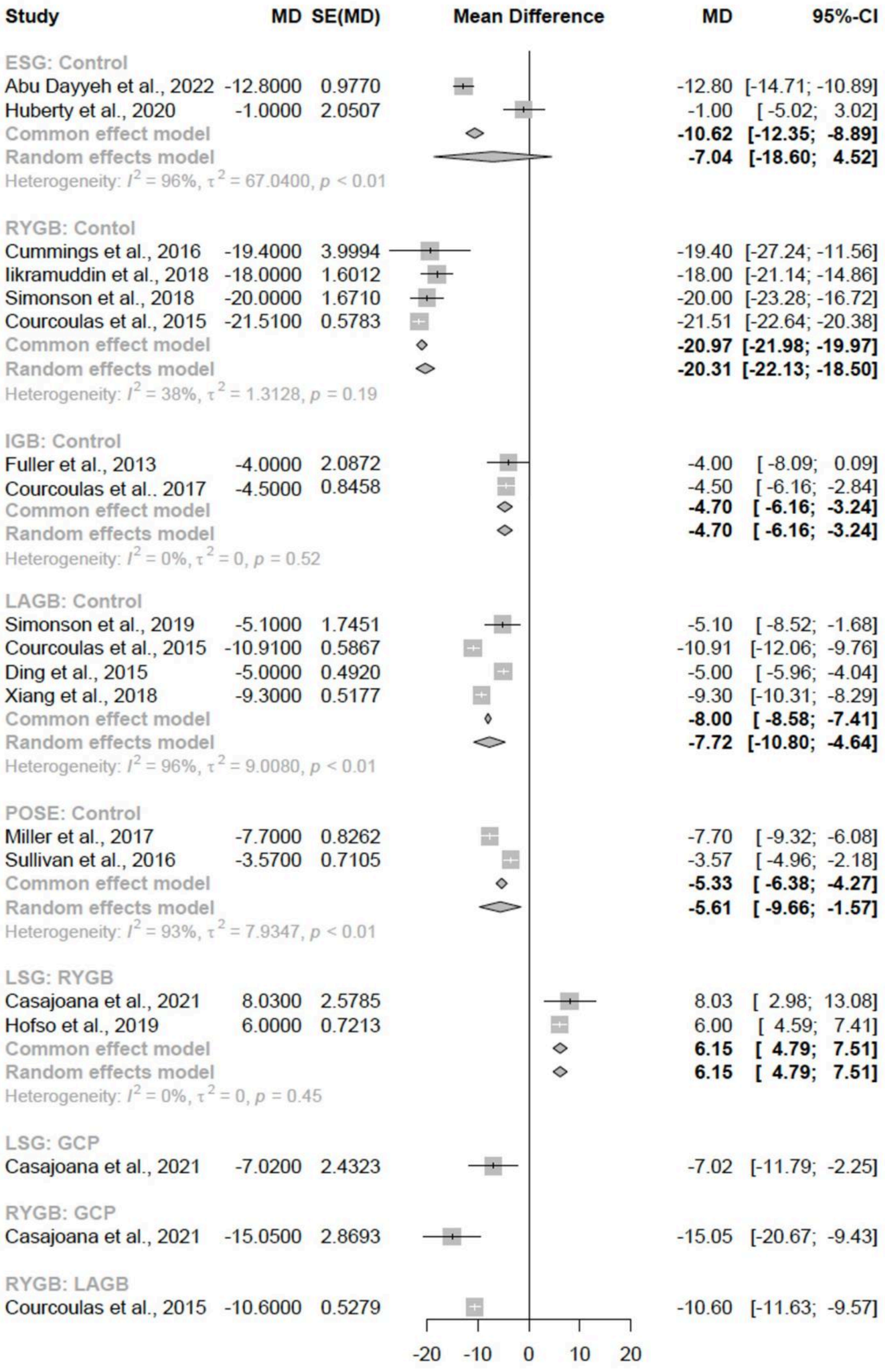

eFigure 7. Direct pairwise comparisons with one intervention versus another or control on HbA1c at endpoint using a random effects model.

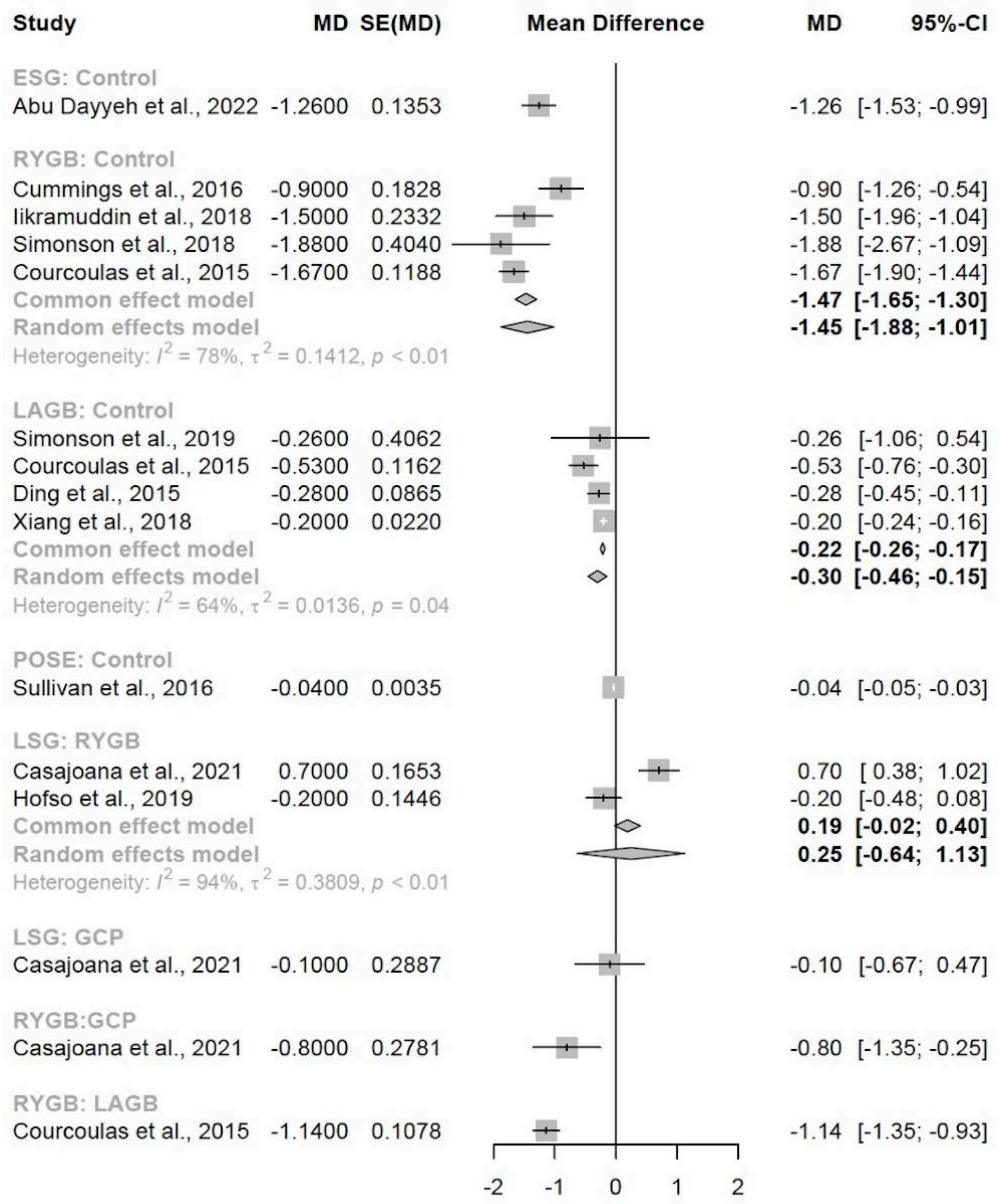

eFigure 8. Direct pairwise comparisons of one intervention versus another or control on T2DM remission at endpoint using a random effects model.

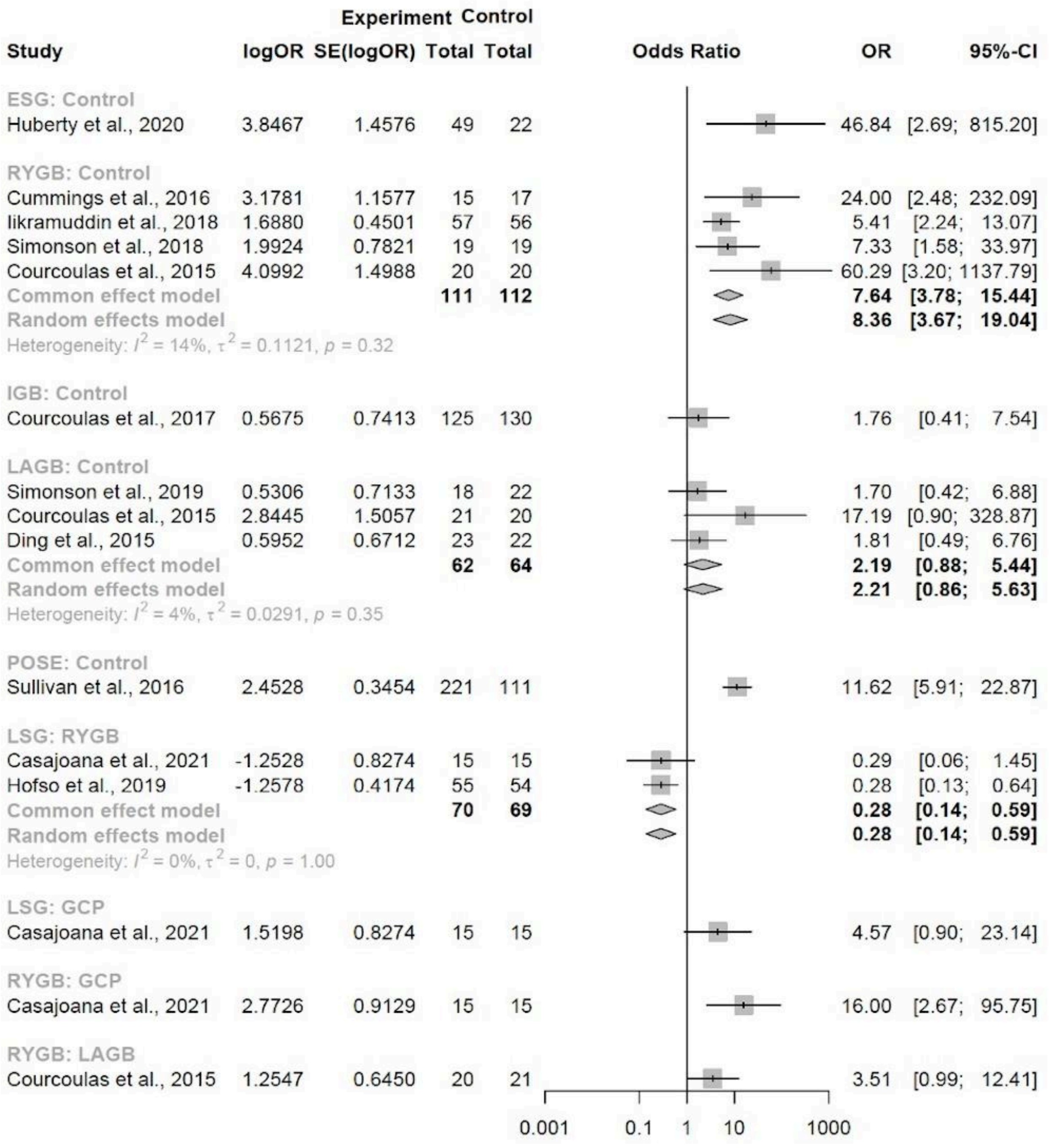

Endosc Int Open | DOI 10.1055/a-2892-8092 | © The Author(s).

eFigure 9. Direct pairwise comparisons of serious adverse events with one intervention versus another or control at endpoint using a random effects model.

Study	logOR	SE(logOR)	Experiment Total	Control Total	OR	95%-CI
ESG: Control						
Abu Dayyeh et al., 2022	2.3574	1.5185	85	124	10.56	[0.54; 207.19]
RYGB: Control						
Abu Dayyeh et al., 2022	3.2488	1.6564	15	124	25.76	[1.00; 661.99]
Simonson et al., 2018	1.5686	1.1714	19	19	4.80	[0.48; 47.68]
Common effect model			**34**	**143**	**8.40**	**[1.29; 54.78]**
Random effects model					**8.40**	**[1.29; 54.78]**
Heterogeneity: $I^2 = 0\%$, $\tau^2 = 0$, $p = 0.41$						
IGB: Control						
Abu Dayyeh et al., 2022	3.2488	1.6564	15	124	25.76	[1.00; 661.99]
LAGB: Control						
Ding et al., 2015	2.3403	1.5225	23	22	10.38	[0.53; 205.27]
Simonson et al., 2019	1.3499	1.6638	18	22	3.86	[0.15; 100.58]
Xiang et al., 2018	1.1213	1.6469	44	44	3.07	[0.12; 77.41]
Common effect model			**85**	**88**	**5.18**	**[0.84; 31.94]**
Random effects model					**5.18**	**[0.84; 31.94]**
Heterogeneity: $I^2 = 0\%$, $\tau^2 = 0$, $p = 0.84$						
POSE: Control						
Sullivan et al., 2016	0.0587	0.4031	221	111	1.06	[0.48; 2.34]
ESG: RYGB						
Abu Dayyeh et al., 2022	-0.8914	1.0166	85	15	0.41	[0.06; 3.01]
ESG: IGB						
Abu Dayyeh et al., 2022	-0.8914	1.0166	85	15	0.41	[0.06; 3.01]
RYGB: IGB						
Abu Dayyeh et al., 2022	0.0000	1.2130	15	15	1.00	[0.09; 10.78]

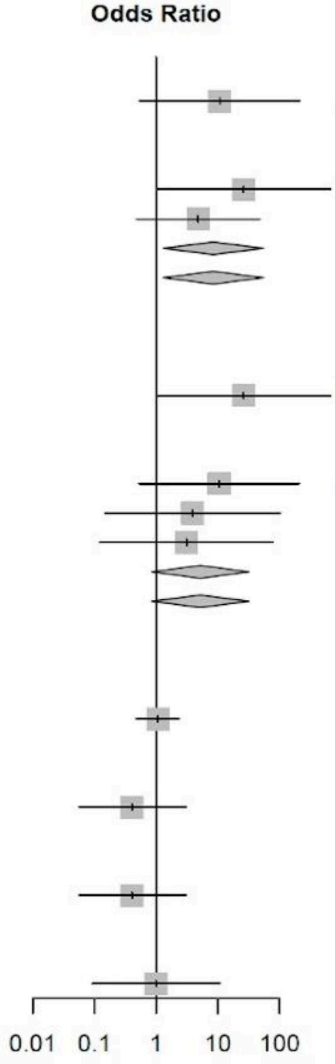

Supplement: Supplementary file 1 — Ergänzendes Material [file 10-1055-a-2892-8092_28930657.pdf]
